# Malonyl-CoA Decarboxylase: A Spotlight on Brain Aspects

**DOI:** 10.3390/brainsci16020220

**Published:** 2026-02-12

**Authors:** Monique Fonseca-Teixeira, Elaine Silva Brito, Clara Beltrao-Valente, Bruna Klippel Ferreira, Patricia Fernanda Schuck, Gustavo Costa Ferreira

**Affiliations:** Laboratório de Erros Inatos do Metabolismo, Instituto de Bioquímica Médica Leopoldo de Meis, Universidade Federal do Rio de Janeiro, Rio de Janeiro 21941-599, RJ, Brazil; monique.teixeira@bioqmed.ufrj.br (M.F.-T.); eln.sbrito@gmail.com (E.S.B.); clara.valente@bioqmed.ufrj.br (C.B.-V.); bruna.klippel@bioqmed.ufrj.br (B.K.F.)

**Keywords:** brain, fatty acid metabolism, malonic acid, malonic aciduria, malonyl-CoA, metabolic disorders

## Abstract

Malonyl-CoA decarboxylase (MCD) is an enzyme that controls malonyl-CoA levels and regulates fatty acid synthesis and oxidation. Although its physiological relevance in peripheral tissues is well known, the role of MCD in the central nervous system remains poorly understood. MCD is expressed in mitochondria, cytosol, and peroxisomes and may be regulated by PPAR-α, AMPK, and SIRT4 in tissues such as muscle, liver and kidney. In the brain, MCD expression varies during development and can respond to nutritional states. Inherited MCD deficiency (malonic aciduria) leads to the toxic accumulation of malonic acid and predominantly affects the central nervous system. The underlying mechanisms leading to brain damage in MCD patients remain unclear. Conversely, pharmacological modulation of MCD activity has been studied in obesity, diabetes, and ischemic injury, highlighting its therapeutic potential. There are still major gaps regarding MCD cellular distribution, regulatory pathways, and metabolic interaction with CPT1c (carnitine palmitoyltransferase 1c) in neural metabolism. A deeper understanding of the role of MCD in brain physiology and pathology may indicate novel therapeutic strategies targeting metabolic disorders that involve altered malonyl-CoA dynamics. Here, we discuss the current knowns and unknowns regarding MCD physiology, regulation, and pathophysiology, emphasizing brain aspects.

## 1. Introduction

Malonyl-CoA decarboxylase (MCD; EC #4.1.1.9) is a key enzyme for the regulation of intermediary metabolism [1,2,3]. MCD is codified by the *MLYCD* gene (located on the chromosome 16q23.3); it is responsible for the decarboxylation of malonyl-CoA to acetyl-CoA [4]. Enzymatic mechanisms to metabolize malonyl-CoA in mammalian tissues were first suggested in 1950 [5]. MCD is important for the metabolism of different tissues, including liver, skeletal muscle, and heart [6,7]. MCD modulation is implicated in the pathophysiology of different metabolic diseases, including obesity and diabetes [8,9,10].

The present review aims at presenting the main roles of MCD in physiology and pathophysiology of diseases, as well as identifying potential areas that could be further explored. Particularly, MCD actions in the brain represent an important avenue to understand brain metabolism and to target diseases affecting these pathways.

## 2. Brain Metabolism

The human brain has a high energy demand, relying heavily on glucose metabolism. Approximately 20% of the oxygen and 25% of the glucose consumed by the human body are dedicated to the brain (despite the brain representing only 2% of the total body mass) [11]. However, the brain is composed of several cell types and regions with specific metabolic profiles, which act both individually and in close cooperation. Among brain cells, neurons and astrocytes are classically known for their interactions and for their important metabolic differences. Complete oxidation of glucose occurs primarily in neurons [12]. Neurons are the main energy consumers, being highly active and requiring a constant energy supply for signal transmission and for excitability maintenance. Astrocytes represent 5% to 15% of the total energy needs of the brain [13]. Neurons and astrocytes present differences in susceptibility to the inhibition of oxidative phosphorylation (OXPHOS). Astrocytes, when facing OXPHOS inhibition, can stimulate glycolysis to prevent ATP decrease [14]. Glycolysis in astrocytes can also result in the production of lactate, which may serve as one of the energy substrates used by neurons [15,16]. The metabolic interaction between neurons and astrocytes is essential for maintaining homeostasis and proper brain function [16].

Glucose metabolism is the primary energy source of the brain, meeting nearly all of its energy needs. However, oxidation of fatty acids and ketone bodies can also contribute to brain bioenergetics [17]. Fatty acid oxidation occurs mainly in astrocytes (but also in microglia) and can contribute up to 20% of the total energy demands of the brain [12,18,19]. There are also certain conditions with low glucose availability when lipids are used as energy substrates, including periods of fasting, strenuous exercise, ketogenic diet, and in some neuropathological conditions [20]. During prolonged fasting, the ketone bodies originating from hepatic metabolism or as a result of ketogenesis in the astrocytes are taken up by neurons and converted to acetyl-CoA, which enters the tricarboxylic acid cycle for ATP generation [21]. Ketone bodies and lactate can cross the blood–brain barrier through monocarboxylate transporters [22].

Lipids and ketone bodies are also needed as an energy source in specific stages of brain development, as in the breastfeeding period [23]. Before birth, glucose is the primary energy source [24,25,26]. After birth, there is a significant shift in the utilization of energy substrates, from glucose to fat. The metabolic environment in the newborn reflects the milk-based diet. In humans, the constitution of breast milk is dynamic and shows high levels of amino acids (colostrum) until the 5th day of lactation. However, protein oxidation is less relevant as an energy source [27]. Mature milk (from the 2nd week of lactation on) is rich in saturated and unsaturated fatty acids [28]. The fat present in mammalian milk constitutes the primary source of calories, comprising approximately 55% of the total caloric content [29]. In addition to mitochondrial beta-oxidation, the brain also breaks down fatty acids by omega-oxidation in the endoplasmic reticulum. In contrast to beta-oxidation, omega-oxidation is not a source of ATP, resulting in the formation of dicarboxylic acids [30].

Oligodendrocytes and microglia also play a crucial role in cerebral energy metabolism [31,32]. Oligodendrocytes can also oxidize ketone bodies and lactate as alternative energy sources, providing energy support for processes such as myelination, maintenance of axonal integrity, and neuronal/synaptic function [32,33,34,35,36,37].

Microglia express all the genes necessary for glycolytic and oxidative metabolism, and the expression of oxidative genes can be comparable to those found in neurons and astrocytes [31]. In inflammatory responses, metabolic reprogramming involves a switch from oxidative phosphorylation (OXPHOS) to glycolysis [18,38]. Microglial homeostasis is crucial for brain health and is closely related to the release of inflammatory mediators in pathological conditions such as aging and neurodegenerative diseases [39,40]. Jian and colleagues demonstrated that depletion of microglia leads to the accumulation of malonyl-CoA and increased fatty acid oxidation in astrocytes [41]. In microglia and macrophages, the expression of pro-inflammatory cytokines favors glycolysis and the production of reactive oxygen species [42,43,44], while anti-inflammatory cytokines induce fatty acid oxidation, contributing to the homeostasis of the neural microenvironment [45]. Moreover, MCD inhibitors can attenuate inflammation in macrophages, suggesting a central role of fatty acids in the inflammatory response [46].

Thus, understanding the MCD role in brain metabolism should consider the different neural cell types and intracellular location, as well as the developmental period.

## 3. MCD in the Physiology of Peripheral and Brain Tissues

### 3.1. MCD Distribution

**Figure 1:**
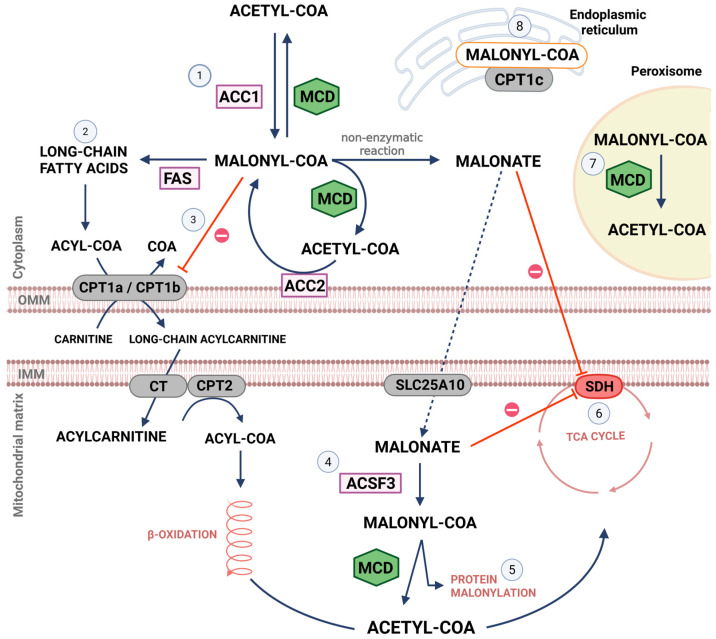
Metabolic pathways involving malonyl-CoA and malonyl-CoA decarboxylase (MCD) in the cytoplasm, mitochondria and peroxisomes. (1) MCD catalyzes the conversion of malonyl-CoA to acetyl-CoA. In the cytoplasm, malonyl-CoA is synthesized by acetyl-CoA carboxylase (ACC1 and ACC2) from acetyl-CoA. (2,3) Malonyl-CoA plays an important role for fatty acid biosynthesis, acting as well as an allosteric inhibitor of the carnitine palmitoyltransferase 1 (CPT1a and CPT1b). (4) In the mitochondria, malonyl-CoA is synthesized by acyl-CoA synthetase family member 3 (ACSF3) from malonate and then it is converted to acetyl-CoA by MCD. (5) Mitochondrial malonyl-CoA can also be important for malonylation of proteins. (6) Malonate is a classic inhibitor of succinate dehydrogenase (SDH) and it crosses the inner mitochondrial membrane through SLC25A10. (7) MCD may be involved in degrading intraperoxisomal malonyl-CoA, which is generated by the peroxisomal β-oxidation of odd chain-length dicarboxylic fatty acids. (8) In addition, malonyl-CoA can also play a role in the endoplasmic reticulum metabolic signaling by its binding to CPT1c (exclusively in the brain). CT, carnitine-acylcarnitine translocase; FAS, fatty acid synthetase; IMM, inner mitochondrial membrane; OMM, outer mitochondrial membrane; SLC25A10, solute carrier family 25 member 10; TCA, tricarboxylic acid cycle. The red arrows with circled minus (⊖) indicate inhibition. Created with BioRender.

MCD has been reported in peripheral tissues, such as skeletal and cardiac muscles, liver, and kidney, and to a lesser extent in the brain [47,48]. MCD activity appears in mammals, birds, bacteria, plants and yeast [49,50,51,52,53,54,55,56]. MCD is a tetramer, and its subunits are linked by disulfide bonds. Each MCD monomer has an N-terminal domain and a C-terminal catalytic domain [57]. The *Mlycd* gene has different promoter regions that are responsible for MCD expression in different cellular compartments, producing organelle-specific isoforms of different molecular weights, including those in cytosol (52–54 kDa), mitochondria (50–51 kDa) and peroxisomes (48–49 kDa) [50,58,59,60] (Figure 1). In the N-terminus of MCD there is a sequence that directs the enzyme to the mitochondria, while in the C-terminus there is a motif that directs the enzyme to the peroxisomes [47].

The subcellular distribution of MCD in different tissues is not completely understood. MCD in rat liver is mainly localized in the cytosol, but it is also found in mitochondria and peroxisomes [47,61]. However, in goose liver it is found exclusively in mitochondria [61,62]. The data on MCD localization in the brain suggest a mitochondrial localization [62,63]. It is still in question whether brain MCD is also present in the cytosol or in peroxisomes. Intramitochondrial MCD affinity for malonyl-CoA differs between liver (Km = 0.04 mM) and brain (Km = 0.5 mM) [63,64]. Interestingly, affinity for malonyl-CoA when recombinant MCD is expressed in *E. coli* also differs when cloned from rats (Km = 0.068 mM) or humans (Km = 0.22 mM) [61,65].

An important area that needs more attention is the localization of MCD in different brain-cell types. In the 90s, the presence of MCD was suggested in some brain cells and regions of adult rats. MCD was reported in neurons and microglia of hippocampus and frontal cortex, and in microglia and Bergman glia in the cerebellum [66].

### 3.2. MCD Regulation

MCD activity and expression can modulate fatty acid metabolism. Inhibition of MCD results in inhibition of fatty acid oxidation by increasing malonyl-CoA levels [67,68,69,70,71]. In addition, increased MCD activity and expression were observed under conditions of increased fatty acid oxidation [48,72,73]. A few mechanisms have been proposed for the modulation of MCD activity, including post-transcriptional and -translational modifications [2,74,75]. MCD can have transcriptional regulation and have its expression increased by peroxisome proliferator-activated receptor-alpha (PPAR-α) in liver and in skeletal and cardiac muscles [74,76,77]. Post-translational modulation of human MCD expressed in Bombyx mori may include the phosphorylation of Ser-204 and Tyr-405 residues. Mutations of these residues lead to decreased decarboxylase activity [78]. Additionally, MCD activity is increased via AMPK phosphorylation during and after exercise in the muscle (especially in those with fast or moderate contraction), liver and adipose tissue [75,79]. MCD can also be regulated by sirtuin 4 (SIRT4), which deacetylates MCD and decreases its enzymatic activity in muscle and adipocyte cell lines [2]. There is no evidence regarding which specific MCD residues are involved in these latter modulations.

In the brain, different nutritional states may impact MCD expression. The role of MCD in the regulation of food intake has been observed in the hypothalamus through mechanisms that involve changes in malonyl-CoA levels and subsequent signaling via CPT1c [80,81,82]. In this scenario, MCD expression is higher in the hypothalamus of fasting animals [81]. In the pituitary gland, MCD expression is positively regulated by resistin, an adipokine that plays crucial roles including the regulation of lipid metabolism, food intake, and gonadal function [83].

### 3.3. MCD During Development

In the brain, the role of MCD is poorly explored, particularly during brain development. The brain develops through a sequence of cellular and functional events that define critical windows of development [84]. Neurogenesis occurs predominantly during the embryonic and early fetal periods, followed by neuronal migration and synaptic formation and refinement. Gliogenesis mainly occurs during late fetal stages, whereas myelination begins in the perinatal period and progresses into adulthood [85,86]. These events must be finely tuned and rely on dynamic metabolic support.

There is an increase in MCD expression in the brain and liver of rats throughout development [66]. In the rat brain, mitochondrial MCD activity progressively increases from the neonatal period into adulthood [48,87]. Whereas brain malonyl-CoA concentrations do not show large variations until adulthood, malonate levels increase with age [88]. Acetyl-CoA, another metabolite important for malonyl-CoA metabolism, has a relatively high expression at birth and slightly decreases with age [88]. The impact of brain MCD expression ontogeny on the levels of these compounds is still to be determined. Despite the lack of information on brain MCD expression during aging, it has been shown that there is increased MCD expression in the gastrocnemius of aged mice [7].

Interestingly, MCD deficiency caused by inherited mutations leads to malonic aciduria, an inborn error of metabolism (IEM) affecting brain structure and function in infant patients [4,89,90]. To date, the molecular mechanisms related to MCD dysfunction are poorly understood.

### 3.4. Malonyl-CoA Metabolism

Malonyl-CoA metabolism is summarized in Figure 1. The synthesis of cytoplasmic malonyl-CoA is undertaken by acetyl-CoA carboxylase. MCD catalyzes the decarboxylation of malonyl-CoA to acetyl-CoA [4]. Malonyl-CoA can be used as 2 carbon units for the biosynthesis of fatty acids by the fatty acid synthase and regulates fatty acid oxidation [91].

The first step in the transport of fatty acyl-CoA across the outer mitochondrial membrane for oxidation is catalyzed by carnitine palmitoyltransferase 1 (CPT1). CPT1 catalyzes the transfer of acyl moieties from acyl-CoA groups (chain length from C12 to C18) to L-carnitine to form fatty acyl-carnitines [92], releasing free CoA. Fatty acylcarnitine is then transported into the mitochondrial matrix in exchange for free carnitine by carnitine-acylcarnitine translocase. Finally, carnitine palmitoyltransferase 2 (CPT2) converts acylcarnitine to fatty acyl-CoA, which is then ready to be oxidized. Mitochondrial beta-oxidation releases acetyl-CoA at the end of every oxidative loop [93]. Acetyl-CoA is a remarkably versatile molecule [94]. Among its actions can be highlighted the fueling of the tricarboxylic acid cycle and therefore ATP production [95].

There are 3 forms of the human CPT1 enzyme: CPT1a, CPT1b, and CPT1c [96,97,98,99]. In the brain, both CPT1a and CPT1b are present especially in the outer mitochondrial membrane [100,101]. CPT1a is more expressed in astrocytes than in neurons, while there is no difference in CPT1b abundance between these cell types [102,103]. CPT1a is also present in microglia and plays a role in neuroinflammation suppression [104]. CPT1a and CPT1b are both inhibited by malonyl-CoA [20]. It has been suggested that only cytosolic malonyl-CoA can inhibit CPT1a and CPT1b, and therefore the oxidation of fatty acids [105].

CPT1c is found in the endoplasmic reticulum, and it is exclusively expressed in neurons [106]. It is found in many brain regions, as well as in the dorsal root ganglia and spinal cord [101,107,108]. Unlike CPT1a and CPT1b, CPT1c has low catalytic activity, using acyl-CoA esters as substrates in the physiological context [101,106,109,110]. Palmitoyltransferase activity of CPT1c is 20 to 300 times lower than that of CPT1a [106] and may contribute to the synthesis of ceramide and sphingolipids [106,111,112]. CPT1c-mediated ceramide synthesis was shown to be associated with appetite-related hormones [80,100,112,113,114,115].

In the brain, CPT1c may be important for dendritic spine maturation during brain development by increasing ceramide levels [111]. CPT1c ablation in mice disturbs synaptic plasticity and may lead to complications of cognitive functions, including learning and memory [116]. In addition, fluctuations of brain malonyl-CoA levels may impact GluA1 trafficking to the plasma membrane in a CPT1c-dependent manner. When CPT1c is devoid of malonyl-CoA, there is a stimulation of phosphoinositide phosphatase suppressor of actin 1 (SAC1) activity. SAC1 has been reported as a protein that regulates vesicular trafficking. Therefore, SAC1 stimulation by CPT1-c disrupts GluA1-containing AMPAr (alpha-amino-3-hydroxy-5-methyl-4-isoxazole propionic acid receptors) trafficking [117]. Hence CPT1c may be considered a strategic target in research related to alterations in the central nervous system.

Malonyl-CoA can also be synthesized in the mitochondria by the acyl-CoA synthetase family member 3 (ACSF3) by binding malonate with CoA [63,87,118]. Malonate is a classic competitive inhibitor of succinate dehydrogenase [119,120] and its main source of production is attributed to the non-enzymatic hydrolysis of cytoplasmic malonyl-CoA. Malonate can enter mitochondria via the dicarboxylate carrier SLC25A10 (solute carrier family 25 member 10), located in the inner mitochondrial membrane [121,122]. Thus, ACSF3 activity lowers intramitochondrial malonate levels, preventing its toxic accumulation [123]. It was hypothesized that mitochondrially synthesized malonyl-CoA can also be important for malonylation of proteins. For instance, malonylation increases the activity of acyl-CoA thioesterase 7 [124], an intracellular enzyme that converts acyl-CoA to free fatty acids and that is highly expressed in neurons [125]. In peroxisomes, malonyl-CoA generated by the peroxisomal beta-oxidation of odd chain-length dicarboxylic fatty acids is catabolized by MCD [61,126]. Additional roles described for malonyl-CoA involve microsomal incorporation into fatty acids used for myelin formation in the brain of young rodents [127].

## 4. MCD in the Pathophysiology of Diseases

### 4.1. MCD Deficiency: An Inborn Error of Metabolism (IEM)

Disorders involving inherited metabolic defects have gained attention in recent years since many patients present a good prognosis if diagnosed early and treated appropriately [128,129]. IEMs are a group of genetic metabolic diseases caused by a deficiency of protein, including enzymes and transporters, affecting multiple metabolic pathways. These defects lead to the accumulation of precursors that can become toxic [130]. The symptomatology is variable, but the central nervous system is frequently affected in IEM [131,132].

MCD deficiency (OMIM #248360), also called malonic aciduria, is an IEM caused by mutations in the *MLYCD* gene. Many pathogenic molecular variations have been associated with the *MLYCD* gene [133,134,135,136,137]. A loss of 30% of MCD activity is sufficient to impact the health of individuals [4,138], with some patients presenting approximately 15% of residual MCD activity [139]. It is not known whether complete ablation of MCD in humans would be compatible with life [133].

MCD deficiency is a disease that affects infants and can lead to neonatal sudden death. It has been reported that some patients may reach adulthood [133]. Newborns and children with MCD deficiency show a variable phenotype. Patients may present feeding difficulties and alterations in skeletal and cardiac muscles (e.g., hypotonia and cardiomyopathy) [89,140,141]. Many signs and symptoms are also frequently associated with neurological damage. Symptoms include seizures [90,133,135,136,137], neurodevelopmental delay (e.g., language and psychomotor impairment, and intellectual disability) [89,135,136,137] and brain magnetic resonance imaging abnormalities (e.g., generalized brain atrophy, white matter abnormalities, and cortical malformation) [90,135,136,142]. So far, the underlying mechanisms of tissue damage in MCD deficiency remain largely unknown, but malonic acid is a potential culprit. Patients with MCD deficiency may present and excrete much higher levels of malonic acid, a biochemical hallmark of the disease [133,139,142,143]. In healthy humans, malonic acid is found in body fluids, including cerebrospinal fluid, blood, saliva, and urine [133,142,144,145,146]. Table 1 shows the levels of malonic acid and MCD activity reported in patients with MCD deficiency and in healthy individuals. A drastic increase in malonic acid levels is also described in MCD knockout mice (approximately 200 times higher) [147]. Interestingly, MCD knockout mice show high early mortality rate, growth retardation, and early cardiac dysfunction [148].

Treatment options for patients with MCD deficiency involve oral levocarnitine administration and dietary management [133,134]. Levocarnitine enhances the synthesis and excretion of acylcarnitines, reducing the accumulation of potentially toxic metabolites and contributing to the restoration of the free CoA pool [149]. It can also improve fatty acid oxidation and attenuate mitochondrial dysfunction, being associated with clinical improvement in patients with MCD deficiency (especially when combined with specific dietary interventions) [149,150]. The dietary treatment often includes a combination of long-chain triglyceride-restricted diet with medium-chain triglyceride-supplemented diet to meet caloric and essential fatty acid requirements in the first few days of life [133,134]. A patient with presymptomatic diagnosis and early treatment showed only mild language and psychomotor delay, with normal cardiac function [151]. However, disease-specific dietary guidelines for MCD deficiency are still unavailable, so more efforts are needed to establish novel and/or more efficient therapeutical avenues for this disease.

**Table 1:** Malonic acid levels and MCD activity in healthy individuals and MCD-deficient patients.

Sample		Healthy Individuals	MCD-Deficient Patients	Ref.
Malonic acid	Blood (µM)	~0.15	~40	[141]
	Saliva (µM)	~0.5	unknown	[145]
	Urine (mmol/mmol creatinine)	<0.1	~104	[132]
	CSF (µM)	~4.5	~180	[141]
MCD activity	Fibroblasts (nmol/h per mg protein)	~8–15	~2–4	[151]

Malonic acid levels in blood, saliva, urine and cerebrospinal fluid (CSF) in health individuals and MCD-deficient patients. MCD activity in health individuals and MCD-deficient patients. MCD, malonyl-CoA decarboxylase.

### 4.2. The Multifaceted Pathophysiological Impact of MCD

MCD impacts different pathophysiological processes. MCD overexpression in the hypothalamus has implications for obesity due to nutritional modulation mediated by the decrease in malonyl-CoA levels, stimulating food intake and progressive weight gain [152,153]. On the other hand, MCD ablation partially prevents the weight gain in rodents subjected to a high-fat diet [154]. MCD manipulation has also been suggested for the treatment of diabetes. Considering the interdependent relationship between the metabolism of glucose and fatty acids, decreased MCD activity and consequent increase of malonyl-CoA levels can favor glucose oxidation (thereby reducing blood glucose levels) [71,154,155]. In addition, MCD pharmacological suppression may be beneficial in the treatment of myocardial ischemia. MCD^−/−^ mice exposed to acute ischemic stress showed a modulation of energy substrate preference (from fatty acids to glucose) in heart, leading to cardioprotection [71,154,155]. Furthermore, MCD inhibition protected cardiomyocytes from insulin resistance induced by lipopolysaccharide, a classic inducer of inflammation [46]. Thus, pharmacological manipulation of MCD activity may be considered a potential therapeutic target for a myriad of metabolic conditions.

## 5. Future Directions

MCD is an important enzyme that contributes to metabolic homeostasis in different tissues. However, many gaps are yet to be filled, particularly in the brain. Having robust understanding of MCD cellular and subcellular distribution in the brain, as well as the elucidation of brain MCD function and regulation, are exciting areas that warrant further research and development. There is an urgent need for future studies using cell-specific approaches to elucidate the role of MCD in each neural cell population for brain physiology and pathophysiology of various diseases. Potential studies include the use of malonyl-CoA biosensors [156] in different brain cell types following genetic or pharmacological manipulation of MCD. These approaches can contribute to the understanding of how intracellular malonyl-CoA levels impact pathways critical for brain homeostasis (e.g., neuroenergetics, brain fatty acids synthesis, CPT1c-driven signaling, etc.). Thus, the role of MCD in the brain needs to be further explored in the context of both health and disease, contributing to the understanding of (i) the physiological roles of this enzyme; (ii) the pathophysiology of MCD deficiency and diseases with altered MCD activity/malonyl-CoA levels; and (iii) the potential therapeutic value of this metabolic target.

## Data Availability

This study is based on previously published literature, and no new data was generated or analyzed.

## Abbreviations

ACC	Acetyl-CoA carboxylase
ACSF3	Acyl-CoA synthetase family member 3
AMPAr	Alpha-amino-3-hydroxy-5-methyl-4-isoxazole propionic acid receptors
AMPK	AMP-activated protein kinase
ATP	Adenosine triphosphate
CT	Carnitine-acylcarnitine translocase
CoA	Coenzyme A
CPT1a	Carnitine palmitoyltransferase 1a
CPT1b	Carnitine palmitoyltransferase 1b
CPT1c	Carnitine palmitoyltransferase 1c
CPT2	Carnitine palmitoyltransferase 2
CSF	Cerebrospinal fluid
FAS	Fatty acid synthase
IEM	Inborn errors of metabolism
IMM	Inner mitochondrial membrane
MCD	Malonyl-CoA decarboxylase
OMM	Outer mitochondrial membrane
OXPHOS	Oxidative phosphorylation
PPAR- α	Peroxisome proliferator-activated receptor alpha
SAC1	Suppressor of actin 1
SDH	Succinate dehydrogenase
SIRT4	Sirtuin 4
SLC25A10	Solute carrier family 25 member 10
TCA	Tricarboxylic acid cycle
